# Preventing mitochondrial reverse electron transport as a strategy for cardioprotection

**DOI:** 10.1007/s00395-023-01002-4

**Published:** 2023-08-28

**Authors:** Hiran A. Prag, Michael P. Murphy, Thomas Krieg

**Affiliations:** 1 Department of Medicine, University of Cambridge, Cambridge, CB2 0QQ UK; 2 MRC Mitochondrial Biology Unit, University of Cambridge, Cambridge, CB2 0XY UK

**Keywords:** Succinate, Reverse electron transport, Malonate, Reactive oxygen species, Mitochondria, Succinate dehydrogenase

## Abstract

In the context of myocardial infarction, the burst of superoxide generated by reverse electron transport (RET) at complex I in mitochondria is a crucial trigger for damage during ischaemia/reperfusion (I/R) injury. Here we outline the necessary conditions for superoxide production by RET at complex I and how it can occur during reperfusion. In addition, we explore various pathways that are implicated in generating the conditions for RET to occur and suggest potential therapeutic strategies to target RET, aiming to achieve cardioprotection.

## Introduction

Despite extensive research, therapies to prevent ischaemia/reperfusion (I/R) injury in myocardial infarction (MI) remain elusive [65–67]. However, one thing is clear: mitochondria play a critical role in the damaging events of I/R injury [21, 42, 43, 52, 177]. Cardiomyocytes rely on mitochondria to oxidise various fuel sources, maintaining the essential ATP/ADP ratio and consuming oxygen in the process, due to their high energetic demands [60, 119]. As the organelles responsible for consuming over 90% of the oxygen intake [156], it is logical that mitochondria play a central role in ischaemic pathologies where oxygen supply to tissues is rapidly depleted [67, 80]. Moreover, mitochondria are not merely ATP producers; they also serve as key metabolic and signalling hubs that underpin (patho)physiology [129], playing a multifaceted and complex role in cardiac I/R injury [42, 101, 120].

Apart from their involvement in the tricarboxylic acid (TCA) cycle as providers of electron transport chain (ETC) cofactors, mitochondrial metabolites intricately interact with the rest of the cell, shaping its phenotype and response to stimuli [33, 55, 129]. This precise interplay becomes particularly evident during ischaemia and reperfusion, defining the response to the shift from homeostasis and ultimately the phenomenon of I/R injury [63, 66, 101]. In this overview, we will explore the mitochondria-centric mechanism that triggers damage in I/R injury by generating the proximal reactive oxygen species (ROS) superoxide by reverse electron transport (RET) and potential therapeutic opportunities in this domain.

## RET in I/R injury pathophysiology

### Thermodynamics of RET in I/R injury

For a long time, it has been hypothesised that a surge of reactive oxygen species (ROS) originating from mitochondria plays a crucial role in I/R injury. Initially, ROS was believed to be a non-specific oxidative event triggered by the reintroduction of oxygen into the ischaemic region [56, 64, 178]. However, more recent research has led to the proposal of a specific mechanistic pathway leading to the production of superoxide [40, 41]. Now, RET at complex I is thought to be the predominant mechanism of the mitochondrial superoxide burst upon reperfusion [40, 41, 121, 173]. This notion is a result of a large body of work detailing the metabolic changes occurring during I/R and their contribution to driving the production of superoxide [40, 41, 113, 141, 173].

As complex I sits close to thermodynamic equilibrium, it is considered to be a reversible catalytic machine, with the direction of electron flow dependent on the protonmotive force (Δp) and relative Coenzyme Q (CoQ) and NADH pool reduction states [3, 121, 176]. This leads to complex I being able to produce superoxide in two catalytic states: forward and reverse electron transport [97, 121, 137]. Superoxide production during forward electron transport (FET) occurs in a situation where the flavin mononucleotide (FMN) group in complex I is reduced. The reduction of the FMN is set by the NADH/NAD^+^ ratio, therefore perturbations in complex I function such as respiratory chain inhibition (e.g. Q-site inhibitor rotenone) or dysfunction lead to an increased NADH/NAD^+^ ratio, driving superoxide production by FET. The second catalytic state of complex I that can produce superoxide is RET [121, 137, 141, 169]. RET occurs when electrons are forced back from the CoQ pool onto FMN and thus able to reduce NAD^+^ to NADH [121, 137, 169].

**Fig. 1:**
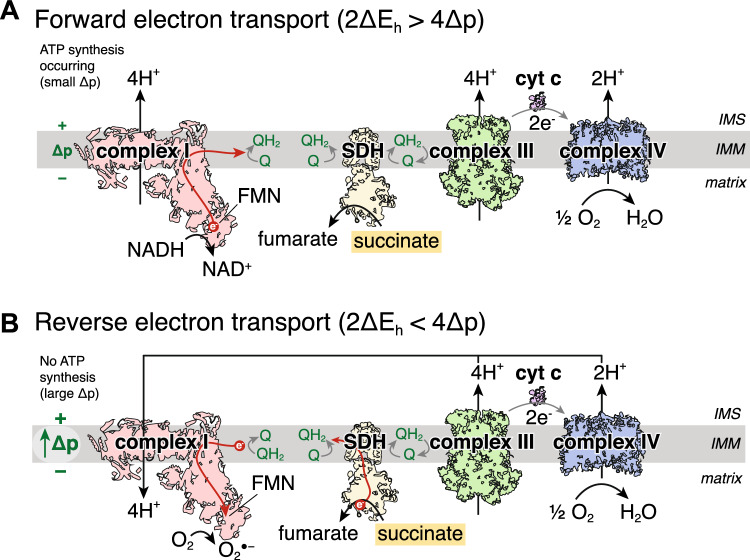
Schematic of conditions required for RET and superoxide production. **A** Complex I operating forward electron transport, oxidising NADH to generate the protonmotive force (Δp) which is used for ATP synthesis. The reduction potential difference between the NAD^+^/NADH and the CoQ/CoQH_2_ redox couples across complex I (ΔE_h_) has to be larger than that required to pump protons across the IMM against the Δp. As 4 protons are pumped per 2 electrons through complex I, for forward electron transport 2ΔEh > 4Δp. **B** In conditions of a large Δp, such as no ATP synthesis, so that 2ΔEh < 4Δp, electrons can be forced backwards from the CoQ pool and onto the complex I FMN which can transfer an electron onto oxygen to produce superoxide. *IMS* intermembrane space, *IMM* inner mitochondrial membrane, *SDH* succinate dehydrogenase, *FMN* flavin mononucleotide, *Δp* protonmotive force, *cyt c* cytochrome c, *O*_*2*_^*●−*^ superoxide, *DIC* mitochondrial dicarboxylate carrier

Thermodynamically, for forward electron transport, the reduction potential difference of the NAD^+^/NADH and CoQ/CoQH_2_ redox couples (ΔE_h_) for 2 electrons to transport across complex I must be large enough to pump 4 protons against the protonmotive force (Δp) across the mitochondrial inner membrane [29, 121]. Therefore, for 2 electrons passing through complex I, 4 protons would be pumped so 2ΔE_h_ > 4Δp (Fig. 1A). For RET, the reverse must be true, in that the reduction potential difference between the NAD^+^/NADH and CoQ/CoQH_2_ pools must be less than the energy required to pump 4 protons across the inner membrane [32, 121, 169]. These conditions would be met in instances of a high CoQ pool reduction state and a large Δp, meaning that 4Δp > 2ΔE_h_, leading to RET through complex I and onto FMN, with either subsequent reduction of NAD^+^ to NADH, or of oxygen to generate superoxide (Fig. 1B) [121, 169]. Furthermore, RET generates the most superoxide out of all the mitochondrial superoxide production mechanisms [121].

For RET to be relevant in I/R injury, we must understand whether the conditions upon early reperfusion are sufficient to drive superoxide production via this mechanism. The low K_m_ of complex IV for oxygen means that upon reperfusion, the oxygen present will rapidly restore electron flow through the ETC [156]. The restoration in ETC activity generates a large Δp as complexes III and IV pump protons across the inner mitochondrial membrane (IMM) [40, 41, 91]. Under normal conditions, the Δp would be utilised by ATP synthase, shuttling the protons back across the IMM and into the matrix to power the generation and release of ATP from ATP synthase, as well as export of ATP to the cytosol by the adenine nucleotide translocator (ANT) [94, 144]. However, during the conditions of early reperfusion, ATP synthesis is limited due to the lack of availability of adenine nucleotides for phosphorylation [40, 41]. This is due to the extensive degradation of the adenine nucleotide pool during ischaemia [40, 106]. Limiting ATP synthesis on reperfusion minimises the utilisation of the Δp across the IMM [153]. Therefore, the metabolic changes during ischaemia seem to be able to satisfy the condition of 4Δp > 2ΔEh for RET to occur (Fig. 1B). On reperfusion, electrons will be transferred from the CoQ pool, through the ETC and onto oxygen. The conditions for RET to occur requires a source of electrons to maintain a highly reduced CoQ pool in early reperfusion [121]. Therefore a substrate that is highly accumulated in ischaemia, rapidly oxidised within mitochondria and feeds electrons directly into the CoQ pool could drive RET at complex I in early reperfusion.

### Succinate at the heart of RET in cardiac I/R injury

A critical finding tying together the idea of ordered RET-derived superoxide production initiating I/R injury is the TCA cycle metabolite succinate. Succinate accumulation in oxygen-compromised environments was well documented in the comparative physiology field [69, 70], however, the link to I/R injury was not made until the precise metabolomic profiling of ischaemic tissues [40]. Succinate was significantly accumulated in numerous ischaemic mouse tissues, including the heart [40]. Furthermore, succinate was one of only three metabolites that accumulated in all the tissue types tested and the only mitochondrial metabolite [40]. The other two metabolites were hypoxanthine and xanthine – both adenine nucleotide degradation products [20, 40, 85, 106]. The many-fold accumulation of succinate in the in vivo ischaemic mouse heart has also been recapitulated in rat, rabbit, pig and human hearts [40, 88, 89, 106], thus succinate accumulation is a conserved hallmark of ischaemia.

**Fig. 2:**
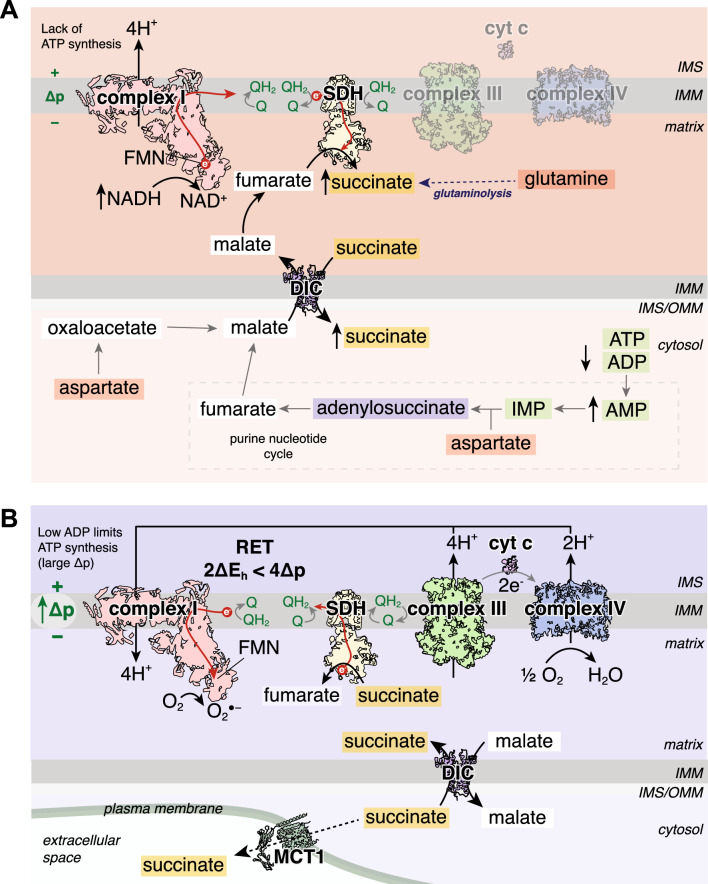
Succinate at the heart of I/R injury **A** Pathways involved in ischaemic succinate accumulation. In ischaemic tissue, succinate accumulation occurs primarily by SDH reversal. This process is driven by the reduced CoQ pool, fed by NADH oxidation by complex I, enabling electrons from the CoQ pool to reduce fumarate to succinate. There are multiple avenues for substrates to lead to succinate accumulation. The degradation of AMP in the purine nucleotide cycle leads to the production of fumarate in the cytosol, which is subsequently converted to malate. The transamination of aspartate to oxaloacetate and subsequent reduction to malate provides another pathway to produce cytosolic malate. This malate can be transported into mitochondria by the DIC in exchange for succinate, also leading to a cytosolic pool of succinate accumulating. The mitochondrial malate can be subsequently dehydrated to fumarate which can feed the production of succinate. Ischaemic succinate accumulation may also occur by the canonical TCA cycle via glutaminolysis. **B** Succinate as a driver of RET on reperfusion. Upon reperfusion, the reintroduction of oxygen restarts the electron transport chain and proton pumping by complexes III and IV. As ATP synthesis is limited due to the degraded adenine nucleotide pool, a large Δp is generated. The CoQ pool remains reduced by the oxidation of the pool of succinate, sustained by the transport of cytosolic succinate into mitochondria by the DIC. Succinate oxidation together with the large Δp drives RET, with electrons from the CoQ pool going on the complex I FMN and onto oxygen, generating superoxide. A proportion of the cytosolic pool of succinate is effluxed from cardiomyocytes via MCT1. *IMS* intermembrane space, *IMM* inner mitochondrial membrane, *SDH* succinate dehydrogenase, *FMN* flavin mononucleotide, *Δp* protonmotive force, *cyt c* cytochrome c, *O*_*2*_^*●−*^ superoxide, *DIC* mitochondrial dicarboxylate carrier, *IMP* inosine monophosphate

The mechanism of succinate accumulation in ischaemia remains uncertain with evidence from varying models pointing to either succinate dehydrogenase (SDH) reversal or canonical TCA cycle activity [38, 40, 151, 174]. SDH is a key node of oxidative metabolism due to its dual role as an enzyme within both the TCA cycle and the ETC. The data from an ex vivo perfused heart model have suggested that ischaemic succinate accumulation occurs predominantly by canonical TCA cycle activity [174]. Here, it was shown that glutaminolysis drives the anaplerotic entry of carbons into the TCA cycle via α-ketoglutarate and that dimethyl α-ketoglutarate could further boost the levels of ischaemic succinate [174]. However, in vivo studies support succinate accumulation occurring due to a reversal in SDH activity, reducing fumarate to succinate (Fig. 2A) [40, 151]. In ischaemia, the CoQ pool becomes highly reduced due to the lack of the terminal electron acceptor oxygen [59, 141]. Thus electrons from cofactors, such as NADH via complex I, reduce the CoQ but can no longer be transferred onto oxygen. Instead, the electrons from the CoQ pool can be used by SDH to reduce fumarate to succinate, with fumarate, therefore, acting as a terminal electron acceptor and succinate accumulating [40, 151]. As no mammalian mitochondrial fumarate transporter has been identified [76] it has been postulated that fumarate transport across the IMM in cardiac mitochondria arises from malate via a partial reversal of the TCA cycle [40, 41]. As malate can be transported freely across the IMM by the mitochondrial dicarboxylate carrier (DIC), this enables numerous cytosolic pathways to converge at malate and then enter mitochondria to enable succinate accumulation (Fig. 2A) [53]. The degradation of the adenine nucleotide pool leads to the production of fumarate via the purine nucleotide cycle (PNC) by the action of adenylosuccinate lyase (ASL) in the cytosol [159]. This cytosolic fumarate can be converted to malate by cytosolic fumarate hydratase (FH) [1, 7]. In addition, aspartate may be an important anaplerotic route driving ischaemic succinate accumulation. Aspartate can feed into the cytosolic production of malate by participating in the PNC as a substrate for adenylosuccinate synthetase [40, 159], or aspartate may be transaminated to oxaloacetate and subsequently metabolised to malate in the cytosol of the ischaemic heart tissue [40, 44].

As malate may be produced in the cytosol of the ischaemic cardiomyocytes, an important step is its transport into mitochondria. Both malate and succinate are substrates for the DIC, thus the cytosolic malate can enter mitochondria in exchange for succinate [53]. This has the dual effect of succinate being diluted in the cytosol preventing the fumarate/succinate ratio from affecting SDH reversal (or maintaining canonical TCA cycle activity [102]), as well as maintaining a supply of malate to sustain succinate production (Fig. 2A) [40]. With the cytosol acting as an electron sink, this enables succinate to accumulate to 2–5 mM as quantified in ischaemic mouse heart tissue [131]. Depending on the conditions and time from the initiation of ischaemia, it may be likely that both canonical TCA cycle and SDH reversal are important in achieving ischaemic succinate accumulation. Nevertheless, regardless of the intricacies of the mechanism, it is clear that succinate is profoundly elevated in ischaemic tissue.

After a short period of reperfusion (~ 1 to 3 min), succinate levels return to those at normoxia [40, 131, 174]. Approximately half of the accumulated succinate effluxes from cardiomyocytes into the circulation via the monocarboxylate transporter 1 (MCT1), while the other half is oxidised by SDH in mitochondria (Fig. 2B) [6, 131, 174]. This rapid oxidation of succinate by SDH provides a source of electrons to maintain a highly reduced CoQ pool during early reperfusion [40, 141]. Together with the large Δp, succinate oxidation maintains a reduced CoQ pool driving RET at complex I and the subsequent superoxide production in early reperfusion (Fig. 2B) [40, 114, 141]. The conditions of early reperfusion are akin to isolated heart mitochondria incubated with succinate in low/non-phosphorylating conditions, where superoxide is maximally produced by RET [121]. Within the reperfused tissue, the cytosolic store of succinate can be transported back into mitochondria via the DIC, sustaining succinate oxidation and returning the succinate levels back to those at normoxia, while driving RET and initiating the damage in I/R injury (Fig. 2B) [41, 53, 121]. With succinate oxidation driving RET and a subsequent burst of superoxide, this initiates the downstream cascade of cell death and injury [40, 80, 95]. The burst of superoxide on reperfusion together with calcium dyshomeostasis and restoration of pH leads to the opening of the mitochondrial permeability transition pore (mPTP) [10, 16, 123]. mPTP opening leads to mitochondrial swelling and rupture activating a series of downstream events resulting in cell death. A number of different mechanisms of cell death have been implicated with I/R, with an initial wave of necrosis and apoptosis in the acute phase of reperfusion (< 24 h) [66]. Later stages of cell death (> 24 h) are now associated with ferroptosis, in an iron-dependent programmed cell death mediated by lipid peroxides [31].

Therefore, the finding of succinate accumulation and oxidation in I/R was instrumental in our current understanding of the ordered production of superoxide upon reperfusion by RET which drives cellular damage and injury.

## Targeting RET therapeutically

As superoxide production via complex I RET is a key initiating event in the pathology of I/R injury, the conditions required to drive RET also provide an opportunity for therapeutic intervention and may help explain mechanistically the basis of cardioprotection of several agents. Below we outline the key factors required for RET-derived superoxide and the current evidence towards pharmacological approaches targeting these to prevent I/R injury.

### Succinate accumulation

The accumulation of succinate during ischaemia is the key to providing both driving forces for superoxide production by RET upon reperfusion: Δp and ΔE_h_. Therefore, targeting the accumulation of succinate during ischaemia is a potential intervention in preventing I/R injury. Indeed, preventing succinate accumulation during ischaemia was a pivotal approach that helped define the importance of succinate metabolism in I/R injury [40]. Succinate accumulation is now seen as a hallmark of ischaemia. Considering the role it plays in driving RET upon reperfusion, interventions that prevent its accumulation may play a role in combatting I/R injury.

**Fig. 3:**
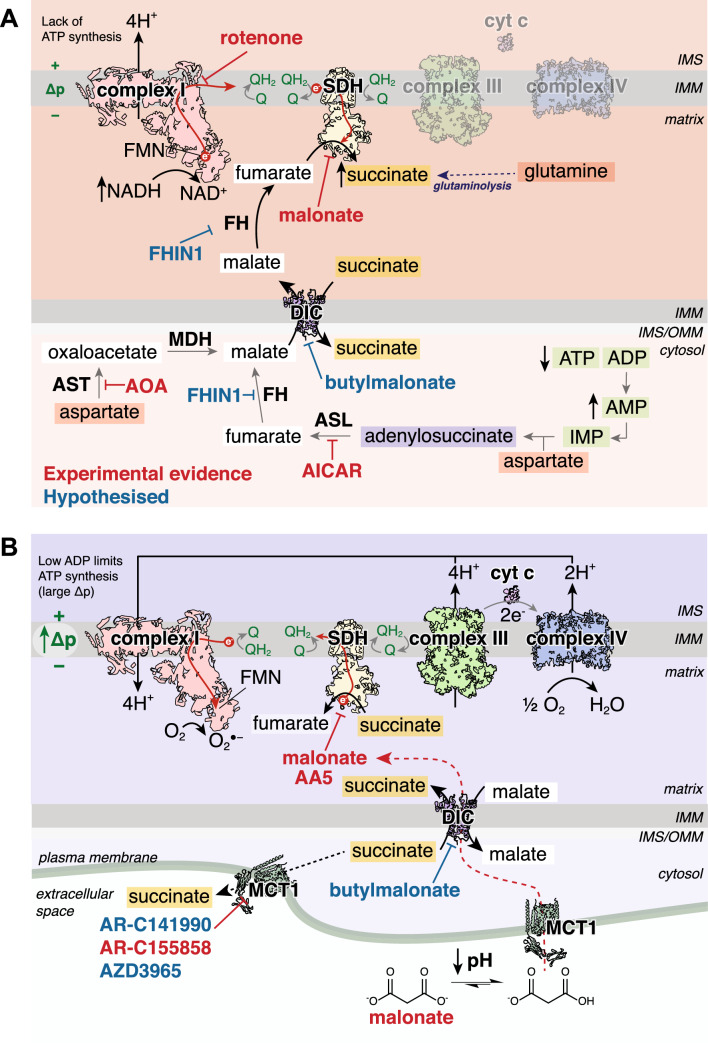
Targeting succinate metabolism to prevent RET. **A** Inhibiting routes to succinate accumulation. Preventing ischaemic succinate accumulation has been shown experimentally by inhibiting SDH reversal with malonate, complex I with rotenone, adenylosuccinate lyase with AICAR and aspartate transaminase with AOA. We hypothesise that inhibiting fumarate hydratase with FHIN-1 or DIC with butylmalonate may also prevent succinate accumulation. Inhibitors in red have experimental evidence of preventing ischaemic succinate accumulation, whereas inhibitors in pink are hypothesised to prevent ischaemic accumulation. **B** Modulating succinate oxidation and transport on reperfusion. Inhibiting SDH with either malonate or AA5 experimentally have shown to be able to prevent RET by slowing succinate oxidation. Malonate selectively enters ischaemic cardiomyocytes on reperfusion due to the low pH protonating malonate into its monocarboxylate form, allowing it to be transported by MCT1. Malonate is subsequently transported into mitochondria by the DIC to selectively inhibit SDH in the at-risk tissue. Blocking succinate efflux via MCT1 with AR-C155858 has been shown to increase superoxide production on reperfusion by RET, due to increasing the proportion of the accumulated succinate oxidised. It is also hypothesised that AR-C141990 and AZD3965 would lead to similar effects in vivo. Targeting the DIC with butylmalonate may also prevent RET by preventing the cytosolic succinate pool from being oxidised by SDH in mitochondria. Inhibitors in red have experimental evidence of modulating RET, whereas inhibitors in pink are hypothesised to affect RET by modulating succinate transport. *IMS* intermembrane space, *IMM* inner mitochondrial membrane, *OMM* outer mitochondrial membrane, *SDH* succinate dehydrogenase, *FMN* flavin mononucleotide, *Δp* protonmotive force, *cyt c* cytochrome c, *O*_*2*_^*●−*^ superoxide, *DIC* mitochondrial dicarboxylate carrier, *IMP* inosine monophosphate, *MDH* malate dehydrogenase, *FH* fumarate hydratase, *ASL* adenylosuccinate lyase, *MCT1* monocarboxylate transporter 1

Preventing succinate accumulation was first found to be a therapeutic option using dimethyl malonate (DMM) [40]. DMM is an ester prodrug of the potent, competitive SDH inhibitor malonate [93, 165], but can freely diffuse across biological membranes and can be hydrolysed by cellular esterases to release malonate in its active form. As SDH reversal leads to succinate accumulation, inhibiting SDH may prevent succinate accumulation during ischaemia (Fig. 3A). When mice were treated with DMM prior to left anterior descending coronary artery (LAD) ligation, succinate accumulation was prevented in the ischaemic tissue [40]. Furthermore, DMM-treated mice also had smaller infarct sizes than control animals [40]. This showed in vivo, not only that SDH reversal contributed to succinate accumulation but also that preventing succinate accumulation during ischaemia may be a promising therapeutic option. In addition, DMM pre-treatment has been used in the isolated Langendorff-perfused heart model with healthy or diabetic rats [78, 158]. Here, DMM led to modest cardioprotection, however, as the levels of succinate and malonate were not measured, it is difficult to ascertain whether SDH was inhibited or whether the diabetic heart affects the metabolism of DMM. However, these results suggest that targeting succinate metabolism may also be of use in treating I/R injury in patients with comorbidities [78, 158]. As well as in MI, DMM has shown utility in preventing cardiac succinate accumulation in organ transplantation [106], haemorrhagic shock [154] and in combination with hypothermic reperfusion [89].

The immune-relevant metabolite itaconate competitively inhibits SDH [98, 116]. The ester prodrug of itaconate, dimethyl itaconate (DMI) was found to be cardioprotective, and its hydrolysis product was presumed to act via the same mechanism as DMM by preventing succinate accumulation [98]. However, it was subsequently found that DMI is not hydrolysed to release itaconate [13, 49, 116]. Rather, the conjugated α-β unsaturation acts as a Michael acceptor and it reacts with free or protein thiols [13, 49, 116] and thus may act similarly to the cardioprotective compound dimethyl fumarate by activating the downstream Nrf2 antioxidant response [7, 92]. Therefore, a key experiment when assessing any potential cardioprotective compound is to measure the levels of the active compound and succinate to assess the impact. It also remains unknown whether Nrf2 activators themselves affect ischaemic succinate accumulation.

As aspartate can feed into at least two different pathways to produce malate within the cytosol of ischaemic tissue, this presents a novel approach which could be targeted therapeutically. Aminooxyacetic acid (AOA) has been used as a pharmacological inhibitor of aspartate aminotransferase (Fig. 3A) [152]. When AOA was infused in vivo, it was able to reduce the levels of accumulated succinate in ischaemic heart tissue [40]. A similar reduction in ischaemic succinate was seen using AICAR (5-aminoimidazole-4-carboxamide ribonucleotide) as an inhibitor of adenylosuccinate lyase (ASL) (Fig. 3A), thus preventing a route for aspartate conversion into cytosolic malate [40]. Both compounds have also been shown to be cardioprotective when infused prior to ischaemia in Langendorff heart models [46, 152]. Although their complete mechanism of action may be complex, their ability to prevent succinate accumulation and thereby prevent RET may have been the reason. A major limitation of both AOA and AICAR is their lack of specificity, with multiple other targets and pathway interactions [147, 164]. Owing to the lack of selectivity, these two compounds may be less useful pharmacological agents to prevent I/R injury for translation, however, there is the potential that other compounds with transient, selective inhibition of either aspartate transamination or adenylosuccinate lyase could be developed.

As fumarate is considered an important intermediate both in the cytosol and mitochondria for ischaemic succinate accumulation [40, 41], targeting fumarate metabolism may be another option to prevent this. As fumarate hydratase (FH) is responsible for the formation of malate in the cytosol to enable its entry into mitochondria, as well as the subsequent production of fumarate from malate in the mitochondrial matrix, FH inhibitors may serve as a strategy to prevent succinate accumulation. FH knockout hearts are protected against I/R injury, which was attributed to the accumulated fumarate leading to antioxidant upregulation via Nrf2 stabilisation [1, 7]. It may well be that FH KO mouse hearts are protected as the lack of FH activity renders the ischaemic heart tissue to be unable to accumulate succinate, however this is currently unknown. FH can be inhibited by the potent, non-competitive drug FHIN-1, which has shown target engagement in vitro and in vivo [37]. With FH localised to both the cytosol and mitochondrial matrix, the effects of FHIN-1 likely represent inhibiting both enzymes, thus may have some mechanistic utility in understanding ischaemic succinate accumulation (Fig. 3A). However, as FH inhibition may be detrimental to cardiac and immune cell function [7, 73], it remains to be seen whether a potent, competitive inhibitor of FH could be developed to transiently inhibit FH to prevent ischaemic succinate accumulation.

Insights from different models may also help bring about new therapeutic targets to prevent ischaemic succinate accumulation. For example, it was recently found that the cardioprotected cyclophilin D (CyD) knockout mouse hearts do not accumulate succinate during ischaemia [132]. While the chronic loss of CyD is responsible for the lack of ischaemic succinate accumulation [132], it may also be of some merit to explore whether chronic cyclosporine A treatment could similarly prevent succinate accumulation, or whether patients taking cyclosporine A chronically for other conditions have smaller infarcts/improved outcomes after MI. Furthermore, comparing the mammalian heart with other species may provide insights into ischaemic succinate accumulation. Anoxia-tolerant animals provide an interesting contrast to mammals, due to their ability to readily experience long periods of anoxia and reoxygenation, without detrimental effects [18]. The succinate levels in the anoxic hearts of red-eared slider turtles (*Trachemys scripta*) are orders of magnitude lower than the ischaemic mouse heart, thus succinate does not accumulate to a level capable of driving RET on reoxygenation [26, 27]. Whether the reduced succinate accumulation is solely due to the colder temperatures or reduced metabolic rate and whether the turtle hearts are similarly protected against ischaemia and not just anoxia remains unknown. Consequently, comprehensive profiling of the pathways involved in preventing succinate accumulation in ischaemic/anoxic tissues may be a useful approach to generate novel therapeutic targets.

While preventing succinate accumulation may be an effective method of preventing I/R injury, its clinical relevance, at least in the context of MI, is debatable. To be effective, a compound capable of preventing succinate accumulation would need to be infused prior to the ischaemic event, which in acute MI is likely to be highly unpredictable. Without this knowledge, the therapeutic window of a compound designed to prevent ischaemic succinate accumulation would be missed. Therefore, compounds preventing succinate accumulation may be more applicable when the period of ischaemia is known, such as elective surgery or organ transplantation when the patient or organ could be pre-treated before the ischaemic event [106]. Furthermore, interventions such as cooling reduce the metabolic rate of the tissue, lessening succinate accumulation during ischaemia [106]; however, how this may be achieved in the setting of a MI may be technically challenging.

### Succinate oxidation

The action of SDH oxidising succinate is also responsible for the rapid return to normoxic succinate levels upon reperfusion [40, 131, 174]. The electrons from succinate help maintain the reduced CoQ pool and drive RET at complex I, leading to the production of the proximal ROS superoxide and the initiation of the damaging cascade in I/R injury. Therefore, approaches to prevent succinate oxidation during reperfusion may attenuate the downstream superoxide production and signalling cascade, preventing I/R injury. Furthermore, targeting succinate oxidation therapeutically represents a highly clinically relevant point of intervention, as a pharmacological agent could be simply administered at the point of removing the vessel occlusion and creating the opportunistic therapeutic window.

The use of the competitive SDH inhibitor malonate was described above in the context of preventing succinate accumulation with its esterified form DMM. DMM was effective at preventing succinate accumulation, however, when DMM was infused at the point of reperfusion, it was not found to be cardioprotective in a murine MI model [134]. The lack of cardioprotection was because hydrolysis of DMM was too slow to achieve sufficient malonate to perturb succinate oxidation by SDH. The rapidly hydrolysable malonate ester prodrug diacetoxymethyl malonate (MAM) [15, 47, 134] could release malonate far more rapidly into cardiomyocytes than DMM and when administered at reperfusion, sufficient malonate was released to reduce succinate oxidation and elicit protection against cardiac I/R injury in vivo [15, 134]. Therefore, competitive inhibition of SDH is a suitable strategy to inhibit RET in a clinically relevant therapeutic window (Fig. 3B), however, an important point is that the drug must rapidly reach its target site in the active form to be effective.

Intriguingly, it was also shown that malonate as the disodium salt (disodium malonate; DSM) was protective against I/R injury in an ex vivo mouse model and in vivo pig MI model [162, 163]. Malonate could enter cardiomyocytes and inhibit SDH on reperfusion leading to reduced superoxide production and smaller infarct size. Curiously, in normoxic mouse tissue infused with malonate, succinate levels only doubled from baseline and were ~ 7 times lower than those achieved during ischaemia, suggesting little SDH inhibition in normoxic tissue [162]. As malonate carries two negative charges at physiological pH, it suggests that malonate uptake into normoxic cardiomyocytes is inefficient, however, can still lead to SDH inhibition and reduced succinate oxidation in I/R [130, 162, 163]. The paradox of malonate’s low cellular permeability, yet robust cardioprotection was recently discovered to be due to malonate’s preferential uptake into ischaemic tissue [130, 146]. In the ischaemic environment, the pH is significantly more acidic, which facilitates the protonation of one of the carboxylate groups of malonate, generating monocarboxylate malonate [130], enabling it to be a substrate for the plasma membrane transporter monocarboxylate transporter 1 (MCT1) (Fig. 3B) [130]. Therefore, malonate can selectively enter the ischaemic tissue upon reperfusion, sparing the healthy tissue where the pH remains unaltered [130, 162]. Once within the cytosol, malonate is rapidly transported into the mitochondria by the DIC, subsequently inhibiting succinate oxidation, superoxide formation and injury [130, 162, 163]. Furthermore, MCT1-dependent malonate uptake could be harnessed to improve the uptake of malonate into the heart tissue by developing a low pH formulation [130]. The low pH malonate formulation increases the proportion of monocarboxylate malonate and leads to lower doses of malonate required for cardioprotection (Fig. 3B) [130]. Another approach which directly targets malonate to mitochondria is via the use of the well-characterised triphenylphosphonium mitochondria-targeting group [143, 150]. Although this approach has been shown to be able to deliver malonate in vivo, whether the levels would be sufficient to prevent succinate oxidation and therefore I/R injury is unknown [133].

The TCA cycle intermediate oxaloacetate is an even more potent competitive inhibitor of SDH than malonate and thus may be an interesting therapeutic candidate for I/R injury [166]. Interestingly, oxaloacetate can also be oxidised by hydrogen peroxide and converted to malonate but whether this process occurs in vivo is unknown [105]. Oxaloacetate has a very high affinity for SDH, with SDH in isolated systems often initially inactive due to endogenous oxaloacetate being bound [135]. However, the understanding of oxaloacetate in biology and its therapeutic use is limited. This may be due to the rapid, spontaneous decarboxylation of oxaloacetate meaning it has a short biological half-life (~ 10 min) [168], as well as being problematic for measurement by the techniques of traditional liquid chromatography-mass spectrometry metabolomics [61]. On top of this, the transport of oxaloacetate into mammalian mitochondria from the cytosol is slow [126]. Therefore, despite oxaloacetate being a very potent competitive SDH inhibitor, its therapeutic utility may be negligible until there are robust methods to accurately measure oxaloacetate in biological samples.

Targeting SDH using the potent, selective, and non-competitive inhibitor atpenin A5 (AA5) has shown cardioprotection (Fig. 3B) [114, 167]. In the Langendorff-perfused heart, when AA5 was infused at reperfusion, it blunted the burst of superoxide upon reperfusion, leading to reduced infarct size, consistent with AA5 preventing RET by blocking succinate oxidation [114]. Although AA5 may be a viable approach in the isolated heart, like other non-competitive inhibitors of SDH, it is unlikely to be a useful approach due to it chronically inhibiting SDH in vivo.

An additional advantage to the use of malonate over other SDH inhibitors is its favourable pharmacokinetics. Malonate has a short plasma half-life (*t*_1/2_ < 40 min, unpublished data), low toxicity and via acidic formulation lower doses can be used to achieve cardioprotection. Malonate is rapidly renally excreted and thereby can limit any off-target effects [145]. Overall, targeting succinate oxidation looks to be a promising approach to targeting RET for cardioprotection.

### Succinate transport

As succinate oxidation is an essential driver of RET, altering the supply of succinate independently of SDH inhibition may provide novel therapeutic targets for cardioprotection (Fig. 3). The current model for succinate accumulation suggests that fumarate acts as a terminal electron acceptor within the mitochondria, being reduced into succinate which can be transported into the cytosol to act as an electron sink [41]. As the succinate concentration would be dramatically diluted in the cytosol, this may facilitate succinate accumulating to high levels in ischaemic heart tissue [40, 131]. Upon reperfusion, there are multiple fates for succinate. To drive RET, succinate is rapidly transported into mitochondria, to enable its SDH-mediated oxidation [40, 131]. However, a proportion of succinate can also leave cardiomyocytes, enter the circulation and thus not contribute to fuelling RET on reperfusion [6, 131, 174]. Therefore, altering the compartmentalisation of succinate may present an attractive option to change the dynamics of the accumulated succinate pool towards RET.

Succinate efflux from the ischaemic heart during reperfusion has been shown to occur in a variety of in vitro and in vivo IR/MI models, including ST-elevated MI patients during primary percutaneous coronary intervention [88, 131, 174]. Approximately 50% of the accumulated succinate pool can efflux from the ischaemic heart tissue [131, 174], thus representing a significant mechanism to rewire the fate of succinate destined for oxidation by SDH. Succinate efflux is driven by the lowered pH of ischaemic tissue, which increases the proportion of the protonated succinate monocarboxylate form [6, 131, 139]. Monocarboxylate succinate can act as a substrate for the monocarboxylate transporter 1 (MCT1; *SLC16A1*), which is highly expressed on the plasma membrane of cardiomyocytes [6, 130, 131]. This provides an outlay for succinate to leave the cardiomyocytes, reducing the amount oxidised by SDH. Despite this efflux of succinate, considerable succinate remains within the cardiomyocytes and is sufficient to drive RET on reperfusion [131]. When MCT1 was selectively inhibited in the Langendorff heart, preventing the release of succinate from the heart on reperfusion, there was an increase in the amount of superoxide produced and damage to the cardiac tissue [114]. This suggests that by blocking succinate efflux from the heart, a greater proportion of the accumulated pool is oxidised by SDH, driving increased RET, superoxide and damage upon reperfusion (Fig. 3B). This result differs from when an MCT1 inhibitor was used in an acute murine MI model, where the inhibitor was found to elicit cardioprotection [131]. This difference may be due to several reasons, including the use of different inhibitors and non-cardiac/off-target effects and the added complexity of the situation in vivo. Nevertheless, both results indicate that targeting cardiomyocyte succinate transport can affect cardioprotection and efforts inhibiting or enhancing this transport may be of use. The impact of other MCT isoforms, genetic variants of MCT1 or MCT1 post-translational modifications on cardioprotection is currently unknown, as well as their expression levels in the heart with comorbidities, which may affect susceptibility to damage.

Succinate compartmentalisation between the cytosol and mitochondria may also play an important role in preventing RET and I/R injury [41]. As succinate from the cytosol fuels RET on reperfusion by re-entering mitochondria, targeting this transport process may be an alternative strategy to disrupt ischaemic succinate accumulation and oxidation on reperfusion (Fig. 3). Succinate transport across the IMM principally occurs via the mitochondrial dicarboxylate carrier (DIC; *SLC25A10*) [53]. The primarily characterised inhibitor of the DIC is butylmalonate [81], however, its potential use in I/R injury is currently unknown. Butylmalonate is a competitive inhibitor of the DIC, but its low potency means high concentrations are required to achieve adequate DIC inhibition [81, 142]. Furthermore, in the I/R injury environment, butylmalonate must compete with the millimolar levels of succinate within cardiomyocytes [59, 131], acting within the cytosol as it cannot enter mitochondria, therefore its utility may be limited until more potent and selective alternatives are developed.

Modulating succinate transport may therefore be an important target in preventing RET by rerouting succinate away from SDH. However, the consequences of succinate leaving cardiomyocytes and entering the circulation are not fully understood. The succinate receptor (GPR91; SUCNR1) is highly expressed on the surface of inflammatory cells such as macrophages, thus succinate may play a role in inflammation in the later phase of I/R injury [82, 104, 131, 160]. Furthermore, succinate-driven RET is also thought to be a key driver for the production of pro-inflammatory cytokines in macrophages [115]. Therefore, the interplay of interfering with succinate transport may be an interesting double-edged sword on one hand preventing RET in cardiomyocytes but increasing the potential negative effects of extra-cardiomyocyte succinate.

### Targeting mitochondrial complex I

**Fig. 4:**
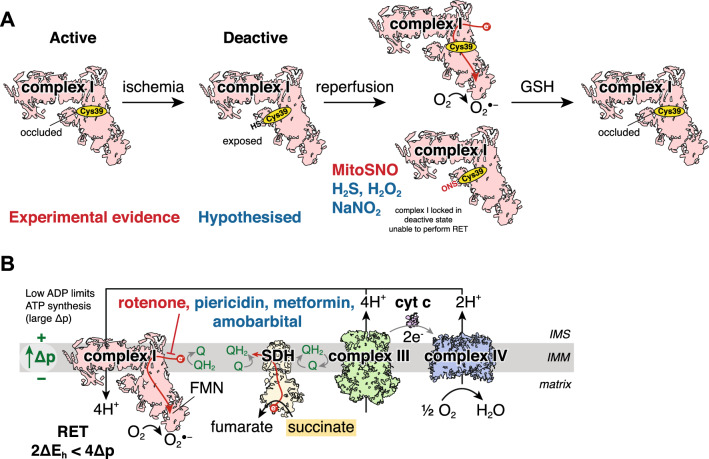
Targeting complex I to inhibit RET. **A** Modulating complex I active/deactive transition. During ischaemia, complex I transitions to a deactive conformation, leading to ND3 cys39 becoming exposed (Cys39-SH). Upon reperfusion, complex I rapidly reactivates, producing a burst of superoxide by RET. Cys39 can be transiently modified by MitoSNO, locking complex I in its deactive state and preventing superoxide production by RET. The modification will be removed from Cys39 by the glutathione or thioredoxin systems, with complex I slowly returning back to its active state. It is also hypothesised that other cysteine modifying agents such as H_2_S, H_2_O_2_ and NaNO_2_ may act by modifying Cys39 to prevent RET. **B** The inhibitor of complex I rotenone has been shown to inhibit superoxide production by preventing RET and can lead to a protective effect. Furthermore, it is hypothesised that other complex I inhibitors such as piericidin, amobarbital and metformin may provide similar cardioprotective effects by preventing RET on reperfusion. *IMS* intermembrane space, *IMM* inner mitochondrial membrane, *SDH* succinate dehydrogenase, *FMN* flavin mononucleotide, *Δp* protonmotive force, *cyt c* cytochrome c, *O*_*2*_^*●−*^ superoxide

As the burst of superoxide upon reperfusion is thought to originate from RET at complex I, complex I remains an important potential target in preventing I/R injury (Figs. 2 and 4) [39, 40]. Additionally, complex I can adopt a catalytically active or deactive state which is important in I/R injury (Fig. 4A) [8, 28]. In conditions where substrate turnover is limited, such as ischaemia, complex I can undergo what is called an active/deactive transition which has been historically defined by the exposure of a solvent-accessible cysteine residue Cys39 on the ND3 subunit of complex I (Fig. 4A) [2]. Alkylation of exposed Cys39 in the deactive state renders complex I catalytically inactive, even when the conditions favouring substrate oxidation return [2, 8, 28]. In this deactive state, complex I cannot perform RET thus transiently keeping complex I in its deactive state during early reperfusion may be a highly targeted method of cardioprotection (Fig. 4A) [28, 45]. This is exemplified by the ND6-P25L mouse where ND6-P25L complex I cannot perform RET but oxidises NADH in the forward catalytic state the same as wildtype [103, 108, 173]. ND6-P25L complex I collapses rapidly into the deactive state due to the mutation creating a disordered Q-site [173]. While forward electron transport can efficiently revert ND6-P25L complex I to the active state, thus catalysing NADH oxidation efficiently, RET conditions cannot consequently complex I remains in its deactive state and RET does not occur [173]. Consistent with RET being a key driver of I/R injury, ND6-P25L mouse hearts are profoundly protected against cardiac I/R injury in vivo, despite all the drivers of RET being present [173].

Compounds capable of transiently modifying Cys39 of ND3 in complex I are attractive strategies to prevent RET and I/R injury. Several compounds that have shown cardioprotection have the potential to modify cysteines, thus there is the possibility that these act by transiently modifying Cys39 on ND3 of complex I to prevent RET (Fig. 4A). An example of this is the mitochondria-targeted S-nitrosothiol MitoSNO which has shown protection in vivo against acute cardiac I/R injury [39, 136] and post-MI heart failure [110]. Mechanistically, MitoSNO has been shown to be able to S-nitrosate the ND3 Cys39 on complex I when administered at reperfusion, locking complex I in its deactive state, preventing RET and affording protection (Fig. 4A) [39]. The modification of Cys39 is transient, with the glutathione and thioredoxin systems capable of reducing the cysteine back to its unmodified form, enabling the complex to switch back to its active form (Fig. 4A) [39, 136]. Similar mechanisms of cysteine modification and RET prevention may be part of the mechanism of protection from several agents that have been previously shown to be cardioprotective including hydrogen sulphide [50], hydrogen peroxide [57], sodium nitrite [148] and other S-nitrosothiols (Fig. 4A) [122]. Furthermore, the active/deactive transition may be relevant in the cardioprotection gained by preconditioning, where multiple rounds of ischaemia and reperfusion sequentially increase the proportion of transiently deactive complex I [90].

The recent finding that complex I ND3 Cys39 can be exposed during active catalysis but labelling has no impact on catalysis raises further questions surrounding molecular characterisation of the active/deactive transition [28]. This suggests that strategies employed to selectively modify deactive complex I may also hit active complex I but not affect its catalytic activity. The subtleties of this remain unknown, however, the significance of active complex I modified ND3 Cys39 in cardioprotection may have implications for RET, cardioprotection and more chronic effects of complex I on pathology. Furthermore, as shown with the recently developed mitochondria-targeted hydrogen sulphide donor MitoPerSulf, it may also be that in part, like hydrogen sulphide, MitoSNO may also transiently inhibit complex IV on reperfusion, reducing respiration and thereby reducing RET-derived superoxide at complex I [111].

As RET requires the flow of electrons from the CoQ pool backwards through complex I, it suggests that strategies to inhibit complex I may be cardioprotective (Fig. 4B). The use of complex I inhibitors, such as the potent and selective inhibitor rotenone, helped distinguish RET as an important mechanism in I/R injury [40, 100]. Under conditions of forward electron transport, rotenone leads to enhanced superoxide production by preventing electrons from NADH reducing the CoQ pool, rather the FMN in complex I will be fully reduced which subsequently reacts with oxygen [121]. However, paradoxically, complex I inhibition prevents superoxide production by RET [121, 137, 169]. Rotenone has been shown to inhibit RET and reduce superoxide production upon reperfusion and therefore may contribute to the mitoprotective effects during ischaemia seen in numerous studies (Fig. 4B) [35, 39, 40, 100, 101], though there is little evidence of rotenone providing cardioprotection after reperfusion ex vivo or in vivo. The reversible, short-acting barbiturate amobarbital can transiently inhibit complex I, thought to be by binding to the same site as rotenone, leading to a significant reduction in infarct size and reduced ROS production on reperfusion [4, 34, 36, 100]. As the amobarbital was added prior to ischaemia, whether this protective effect is by inhibiting RET on reperfusion, or alternative effects during ischaemia is unknown. Furthermore, complex I inhibition has also been shown to prevent succinate accumulation in isolated mitochondria under pseudo-ischaemic conditions (Fig. 3A) [174]. As electrons from NADH feed into the CoQ pool via complex I during ischaemia and can be used to reduce fumarate to succinate, inhibiting complex I with rotenone may prevent this by attenuating succinate accumulation. Therefore, rotenone may play a dual role in cardioprotection, however, the prevention of succinate accumulation by complex I inhibition in tissues and in vivo is unclear.

A limitation of many of the complex I inhibitors is their dose-limiting toxicities [77, 172, 175]. As complex I inhibitors often bind non-reversibly to the Q-site of complex I, their hydrophobicity increases membrane permeability and potency, enhancing off-target effects [172, 175]. As such, the translatability of such compounds has recently been questioned after unfavourable human trials [17, 77, 172]. Biguanides, such as metformin, can inhibit complex I [24, 25] and intriguingly prefer binding complex I in its deactive state when tested in vitro [107]. Metformin has shown protection against acute cardiac I/R injury in MI when high doses were administered at reperfusion [14, 125]. The mechanism of protection by metformin is likely to be highly complex due to its utility in many pathways, however, if metformin can inhibit complex I, inhibiting RET may be part of this cardioprotective mechanism. The current evidence for metformin inhibiting complex I in vivo is limited [68, 157]. As metformin is a hydrophilic, positively charged compound and requires high concentrations to achieve substantial complex I inhibition [24, 25], there are many barriers limiting its effectiveness [54]. While the organic cation transporter 1 (OCT1) that transports metformin is present on the surface of cardiomyocytes [58], how and the rate at which metformin enters mitochondria is unknown [54]. Therefore, despite metformin representing a hydrophilic, highly tolerated and clinically approved drug, many unknowns of the action of metformin on mitochondria exist.

Overall, complex I may be an important druggable target to prevent RET for cardioprotection. As complex I is important for cardiac mitochondrial function, interventions must be transient to enable inhibition of RET on reperfusion, yet subsequently allow for normal respiration. Targeting RET selectively, such as transiently modifying ND3 Cys39 in deactive complex I is an attractive prospect, with endogenous mechanisms to reverse the modification and relieve complex I inhibition. However, it remains to be seen whether these compounds have detrimental off-target cysteine modifications and their translatability in large animal models and humans.

### Manipulating the mitochondrial membrane potential

**Fig. 5:**
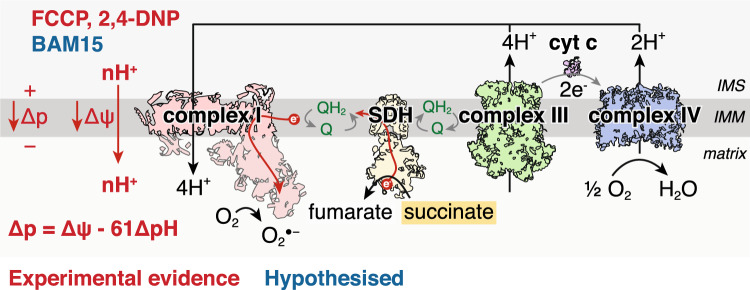
Manipulating mitochondrial membrane potential to prevent RET. Uncoupling the mitochondrial membrane potential with uncouplers FCCP and 2,4-DNP can prevent superoxide production by RET in I/R injury. By short-circuiting the IMM, protons flow from the IMS to the matrix and therefore reduce the Δψ. As Δψ is a large proportion of Δp, (Δp = Δψ – 61ΔpH; given that Δp ~ 180 mV and ΔpH ~ 0.5 U) small changes in Δψ will significantly affect Δp and thereby the driving force for RET to occur. It is also hypothesised that BAM15 may be able to similarly prevent RET from occurring due to its selective uncoupling of the inner mitochondrial membrane. *IMS* intermembrane space, *IMM* inner mitochondrial membrane, *SDH* succinate dehydrogenase, *FMN* flavin mononucleotide, *Δp* protonmotive force, *Δψ* mitochondrial membrane potential, *cyt c* cytochrome c, *O*_*2*_^*●−*^ superoxide

A large Δp across the mitochondrial inner membrane is important for superoxide production via RET. As mitochondrial membrane potential (Δψ) is a large component of Δp (Δp = Δψ—61ΔpH [124]), methods which can reduce mitochondrial membrane potential during early reperfusion may be protective against I/R injury (Fig. 5). By interfering with the mitochondrial membrane potential and short-circuiting the ETC, by providing a route for protons to re-enter the mitochondrial matrix, this leads to oxidation of the CoQ pool, thus nullifying another driver of RET.

The importance of mitochondrial membrane potential in I/R injury is highlighted by studies showing cardioprotection dependent on uncoupling protein 1 (UCP1) [71, 74]. UCP2 and UCP3 have been implicated in protection against cardiac I/R injury, however, their mechanism of action is independent of mitochondrial membrane potential uncoupling [30]. Several previous studies have shown the cardioprotective effects of pharmacological uncouplers, however, their mechanisms of action were determined to be due to impacting mitochondrial membrane potential-dependent ion channel function, rather than electron transport and the CoQ pool directly [22, 23, 30]. FCCP was shown to be cardioprotective and mechanistically initially was thought to afford protection like preconditioning by affecting the mitochondrial K_ATP_ channel [30]. Low doses of FCCP infused into isolated hearts or treatment of ventricular cardiomyocytes were cardioprotective, independent of effects on K_ATP_ channel activation [23]. However, these effects were also independent of membrane potential changes detected by TMRM, though the dynamic range of TMRM may not have been sufficient to detect the membrane potential changes [22]. Furthermore, when isolated rat hearts were reperfused with 2,4-dinitrophenol (DNP), this led to a presumed block in mitochondrial calcium entry and cardioprotective effect [51]. As RET is highly sensitive to small membrane potential changes, it is likely that even these concentrations of uncoupler RET would be affected and may be responsible for the cardioprotective effects seen (Fig. 5) [117].

A key limitation of the ‘classical’ mitochondrial uncouplers is their lack of discrimination for mitochondrial membranes leading to uncoupling of the plasma membrane as well. Impacting the plasma membrane proton gradient may have a multitude of effects, including affecting lactate transport by MCT1 which may provide a secondary mechanism of action of these uncouplers in cardioprotection. Furthermore, as pH plays a critical role in opening of the mPTP, alteration of mitochondrial and cytosolic proton flux may play a key part in the protective mechanism independently of the effects on the respiratory chain. The recently developed mitochondria-membrane selective uncoupler BAM15 has shown potent uncoupling activity, without changes in plasma membrane ion movement [83]. The mechanism of the selectivity of BAM15 for the mitochondrial inner membrane is unknown, however, it has been postulated that the lower mitochondrial pH or difference in lipid composition may be involved [83]. The BAM15 has shown a multitude of beneficial effects in various disease models including renal I/R injury [83], sepsis [161] and diet-induced obesity [5], thus it may have utility against cardiac I/R injury.

While a limitation of the use of uncouplers has been their questionable safety profile, with DNP leading to several fatalities [127], the toxicity of these compounds may be somewhat mitigated in their use against cardiac I/R injury due to their single use. Also, liver-targeted and controlled-release formulations of DNP have significantly reduced toxicity [127, 128]. Low doses of selective uncouplers at reperfusion may transiently depolarise mitochondria and prevent RET on reperfusion without long-term undue toxicity.

### Adenine nucleotide pool degradation

**Fig. 6:**
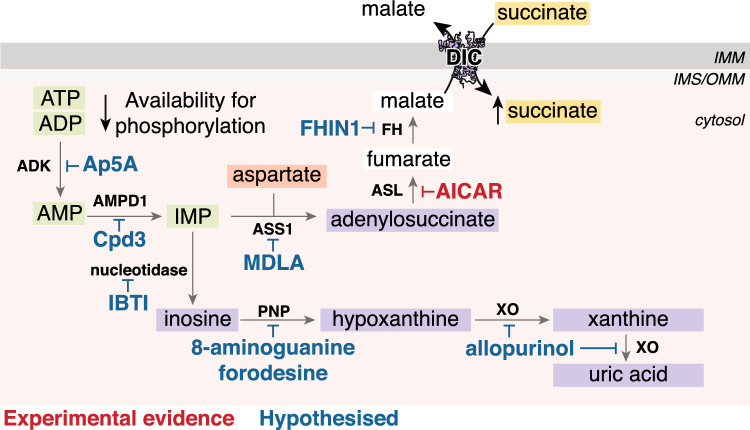
Adenine nucleotide pool degradation targeting to prevent RET. Preventing adenine nucleotide pool degradation, may prevent RET by a dual mechanism. Firstly, as shown with AICAR inhibition of ASL [40] (or hypothesised by FH inhibition by FHIN-1 [155] or ASS1 inhibition by MDLA [75]), targeting this pathway may prevent succinate accumulation during ischaemia, thus prevent succinate oxidation driving RET on reperfusion. Otherwise, it may be hypothesised that inhibitors such as Ap5A [96] which inhibits ADK may prevent ADP breakdown to AMP, thus maintaining ADP levels to allow ATP synthesis using the Δp and thus preventing RET. Other hypothesised targets and inhibitors of the adenine nucleotide degradation pathway include: AMP deamination to IMP (AMPD1 inhibition by Cpd3 [75]), IMP hydrolysis to inosine (nucleotidase inhibition by IBTI [149]), hydrolysis of inosine to hypoxanthine (PNP inhibition by 8-aminoguanine [19] or forodesine [12]) or hypoxanthine oxidation to xanthine and subsequently to uric acid (XO inhibited by allopurinol [85]). These interventions may be hypothesised to maintain the adenine nucleotide pool intermediates and expedite resynthesis of the adenine nucleotide pool. *ASL* adenylosuccinate lyase, *ASS1* adenylosuccinate synthase 1, *MDLA* α-methyl-DL-aspartic acid, *ADK* adenylate kinase, *IMP* inosine monophosphate, *AMPD1* AMP deaminase 1, *PNP* purine nucleoside phosphorylase, *XO* xanthine oxidase

It was previously thought that ATP is rapidly depleted during ischaemia, leading to a large pool of ADP readily available for ATP synthesis upon reperfusion [11, 84]. More recently, it has become apparent that during ischaemia adenylate kinase catalyses the conversion of 2 ADP to ATP and AMP, thus maintaining a source of ATP in non-oxidative conditions [40, 79, 106]. This leads to elevated AMP, which is consequently degraded, with large elevations in xanthine and hypoxanthine as remnants from their adenine nucleotide past (Fig. 6) [40, 106]. The depletion of the ADP pool is an important aspect of the ability of mitochondria to produce superoxide via RET. A depleted ADP pool in ischaemic tissue reduces the ADP available for phosphorylation upon reperfusion, thereby preventing the Δp from being used for ATP synthesis [121]. High mitochondrial Δp is a prerequisite for RET to occur, thus using the Δp for ATP synthesis reduces its magnitude across the IMM and prevents RET. This has been shown experimentally in isolated heart mitochondria respiring on succinate, where the addition of ADP reduces the levels of RET-derived H_2_O_2_ [113]. Therefore, preventing the depletion of the adenine nucleotide pools to sustain ADP levels may be another strategy for preventing RET.

Several reports have suggested the cardioprotective effects of kynurenic acid, however, the mechanism of protection has been poorly understood [109, 140, 170]. Recently, it has been proposed that a key part of the protection of kynurenic acid is through salvaging the ATP pool, preventing ATP hydrolysis by reverse action of ATP synthase, during ischaemia [170]. Here, kynurenic acid is thought to act as a ligand of GPR35 on the plasma membrane [112], with the receptor subsequently internalised and trafficked to the mitochondrial outer membrane [170]. The internalised GPR35 is postulated to be able to inhibit mitochondrial adenylate cyclase, prevent protein kinase A phosphorylation of ATP synthase inhibitory factor subunit 1 (ATPIF1) and thereby promote ATP synthase dimerisation, preventing ATP hydrolysis [170]. The details of how GPR35 enacts its effects at the level of the mitochondrial matrix, despite binding at the outer mitochondrial membrane remain incomplete. Further investigation into whether activation of GRP35 prevents degradation of the adenine nucleotide pool, and not just ATP, is warranted as this aspect remains unclear. If degradation of the adenine nucleotide pool is prevented, this may lead to increased ADP levels in the ischaemic tissue, which may provide a rapid means to reduce the Δp via its phosphorylation by ATP synthase and prevent RET in early reperfusion. Despite this, the actions of kynurenic acid on the ATP pool suggest that strategies to prevent ATP hydrolysis and subsequent adenine nucleotide pool degradation may be effective at preventing cardiac I/R injury.

Many studies have interfered with the adenine nucleotide degradation pathway genetically and have shown their impact on cardioprotection [20, 62, 72, 85, 138]. However, historically these often have used conventional knockout of the gene from conception in the mice, which may lead to the upregulation of compensatory pathways to maintain an appropriate ATP/ADP ratio and thus complicate the interpretation and relevance. Several drugs targeting enzymes within the adenine nucleotide degradation pathway show protection against cardiac I/R injury [40, 41, 85, 138]. Their cardioprotective effects have mostly been attributed to their prevention of oxidative stress, such as with allopurinol inhibiting xanthine oxidase superoxide production [85], however, an alternative mechanism could be via their interference with adenine nucleotide breakdown [20, 85]. Preventing the complete breakdown of adenine nucleotides during ischaemia may facilitate their salvage and synthesis into adenine nucleotides upon reperfusion. By more rapidly re-synthesising the adenine nucleotide pools, this may again lead to utilising the Δp and preventing RET. Figure 6 highlights pharmacological inhibitors of the adenine nucleotide degradation pathway, which may be hypothesised to affect RET. Despite the potential to prevent the complete breakdown of the nucleotide pool, whether this impacts RET directly remains unknown. Also, the cardioprotection by compounds targeting certain enzymes of the adenine nucleotide degradation pathway is poorly characterised, thus further work may be required in this area to understand its relevance. Another caveat to targeting adenine nucleotide degradation is the requirement for treatment prior to the onset of ischaemia, therefore even if interventions may be able to lead to conditions able to prevent RET, they are less likely to be suitable for acute MI.

Furthermore, targeting adenine nucleotide pool degradation may also impact the accumulation of succinate, considering it is thought to be an anaplerotic source of substrate for SDH reversal to produce succinate (Fig. 6) [40, 41]. The rerouting and regeneration of AMP in the PNC leads to the production of fumarate via ASL, as detailed above (see Sect. "Succinate accumulation" and Fig. 3A). This fumarate is converted to malate by fumarate hydratase, which can be transported into mitochondria and act as a precursor to produce succinate during ischaemia. Therefore, affecting the pool of adenine nucleotides may subsequently affect the flux through the PNC and subsequent production of succinate, via the PNC-derived fumarate.

The degradation of adenine nucleotide pools is a hallmark of ischaemia and may impact both the accumulation of succinate and the conditions required to drive RET. Little direct evidence exists as to whether interventions at the nucleotide pool level alter succinate accumulation or can prevent RET, thus future insights into the usefulness of targeting this pathway may be warranted.

## Conclusions and future perspectives

Overall, RET-derived superoxide upon reperfusion is an important initiator of the damage in I/R injury. The extreme conditions of ischaemia remodel cardiac metabolism and lead to the environment satisfying the thermodynamic requirements for RET to occur on reperfusion. Therefore, targeting the pathways which enable the conditions for RET to occur may be an effective strategy to minimise cardiac I/R injury (Table 1). Furthermore, due to the far-reaching changes in metabolism in ischaemia which help support RET upon reperfusion, it may be that several drugs historically found to be cardioprotective may have acted by affecting succinate accumulation or preventing RET-derived superoxide production. Currently, targeting succinate oxidation with malonate seems to have the greatest experimental evidence, with protection in small and large animal models and ischaemia-selective properties. Malonate has also shown effectiveness in resuscitation [171], ischaemic stroke [118] and cardiac regeneration [9] and thus may help break the cycle of failures in preventing cardiac I/R injury. Despite this, history tells us that many further studies are required for successful translation from the bench to the clinic in this field [21, 65, 99, 146]. Currently, there is no data surrounding the use of malonate in human myocardial tissue and whether the same mechanisms from animal models are applicable [48, 86, 87]. Furthermore, there is a lack of evidence of systemic malonate administration and cardioprotection in large animal models [86]. Also, how malonate may affect the normal physiology of conscious large animals needs to be addressed [99]. Aside from malonate, many questions remain for all these therapeutic approaches, in particular: is targeting RET effective in conjunction with comorbidities, are there any gender differences in the protection, are these interventions still effective in aged animals and how does inhibiting RET translate to long-term outcomes? Even though many questions remain open, the orthogonal approaches targeting RET and leading to cardioprotection are promising that RET is a worthwhile target to prevent cardiac I/R injury.

**Table 1:** Summary of experimental evidence of cardioprotection of pharmacological agents which may impact RET

	Inhibitor	Proposed mode of action	Species and Model	Evidence for impact on RET	End-point assessed	References
Ischaemic succinate accumulation	Dimethyl malonate (DMM)	SDH inhibition	C57BL/6J mouse LAD ligation	↓ ischaemic succinate, ↓ ROS on reperfusion (MitoB)	↓ TTC- infarct size (2 h reperfusion)	[40]
			Zucker diabetic fatty (ZDF) rat and non-diabetic control Langendorff global ischaemia	–	↓ TTC- infarct size (2 h reperfusion)	[78, 158]
			New Zealand white rabbit LAD ligation	–	↓ TTC- infarct size (3 h reperfusion)	[89]
			Human heart arterial strips (simulated ischaemia)	–	↑ Contractile force recovery	[78]
	Rotenone	Complex I inhibition	C57BL/6J mouse Langendorff global ischaemia	↓ ischaemic succinate	–	[174]
	AOA	Aspartate transaminase inhibition	C57BL/6J mouse LAD ligation	↓ ischaemic succinate	–	[40]
			Wistar rat Langendorff global ischaemia	–	↓ TTC- infarct size (2 h reperfusion)	[152]
	AICAR	Adenylosucc-inate lyase inhibition	C57BL/6J mouse LAD ligation	↓ ischaemic succinate	–	[40]
			C57BL/6J mouse Langendorff global ischaemia	–	↓ TTC- infarct size (30 min reperfusion)	[46]
Succinate oxidation	Disodium malonate (DSM)	SDH inhibition	C57BL/6J mouse Langendorff global ischaemia	↓ succinate oxidation	↓ TTC- infarct size (2 h reperfusion)	[162]
			Hybrid farm pig LAD ligation	↓ succinate oxidation	↓ TTC- infarct size (2 h reperfusion), no change in haemodynamic parameters or regional myocardial function	[163]
			C57BL/6J mouse LAD ligation	↓ succinate oxidation, ↓ ROS on reperfusion (MitoB)	↓ TTC- infarct size (2 h reperfusion)	[130]
	AA5	SDH inhibition	C57BL/6J mouse Langendorff global ischaemia	↓ mitochondrial superoxide on reperfusion (MitoSOX)	↓ TTC- infarct size (1 h reperfusion)	[114, 174]
Succinate transport	AR-C155858	MCT1 inhibition	C57BL/6J mouse Langendorff global ischaemia	↑ mitochondrial superoxide on reperfusion (MitoSOX)	↑ TTC- infarct size (1 h reperfusion)	[114]
	AR-C141990	MCT1 inhibition	C57BL/6J mouse LAD ligation	–	↓ TTC- infarct size (2 h reperfusion)	[131]
Targeting complex I	MitoSNO	Alkylation of ND3 Cys39 on complex I	C57BL/6J mouse LAD ligation	↓ ROS on reperfusion (MitoB)	↓ TTC- infarct size (2 h reperfusion) ↓ MRI-assessed post-MI HF (28 days reperfusion)	[39, 110]
	Amobarbital	Complex I inhibition	Fischer-344 rat Langendorff global ischaemia	–	↓ TTC- infarct size (30 min reperfusion)	[36]
			Guinea pig Langendorff global ischaemia	↓ superoxide on reperfusion (Dihydroethidium)	↓ TTC- infarct size (2 h reperfusion)	[4]
	Rotenone	Complex I inhibition	Sprague–Dawley rat primary adult cardiomyocytes	↓ superoxide on reperfusion (Dihydroethidium)	–	[40]
Mitochondrial Δψ	FCCP	Uncouple IMM	Wistar rat Langendorff global ischaemia	–	Increased recovery of left ventricular developed pressure on reperfusion (40 min reperfusion)	[23]
	2,4-DNP	Uncouple IMM	Sprague–Dawley rat Langendorff global ischaemia	–	Delayed calcium accumulation and loss of sarcolemmal integrity (30 min reperfusion)	[51]

## Data Availability

There is no data associated with this manuscript.
